# The Results of Fetal Chondrocytes Transplantation in Patients with Rheumatoid Arthritis

**DOI:** 10.5195/cajgh.2014.164

**Published:** 2014-12-12

**Authors:** Natalya Krivoruchko, Saltanat Tuganbekova, Gulnar Rakhimbekova, Karlygash Kuzembaeva, Lina Zaripova

**Affiliations:** National Research Medical Center, Astana, Kazakhstan

**Keywords:** rheumatoid arthritis, fetal chondrocyte transplantation, chondrocytes

## Abstract

**Introduction:**

Nowadays anti-inflammatory and immunosuppressive therapy has significantly improved the quality of life and prognosis of rheumatoid arthritis (RA). Nevertheless, there are still many patients with progressive rheumatoid inflammation, resulting in the destruction of joints. Cell therapy seems like a promising direction in rheumatology. The aim of our research was to evaluate the efficacy of fetal chondrocyte transplantation in patients with RA.

**Methods:**

We examined 60 patients with rheumatoid arthritis (I – III stages) between 20 and 63 years of age. They were divided into 2 groups: the first group underwent the fetal chondrocytes transplantation (n = 40), and the second was a control group who got conservative therapy (n = 20). Donor cells were taken from the chondrogenic layer of the humerus or femur heads and hip condyles of human embryos in gestation for 17–20 weeks. A suspension of fetal chondrocytes injected into affected areas of the articular surfaces under X-ray control. Cell viability was determined before the injection. Efficacy of the therapy was assessed by clinical, instrumental, and laboratory tests. *This clinical trial was allowed by The Ministry of Public Health and Ethics Committee.* All of our patients gave informed consent for the fetal chondrocytes transplantation.

**Results:**

Evaluation of the clinical manifestations of RA in the first group of patients showed 3.7 times decrease in pain and 1.6 times relief of synovitis. Complete reduction of contracture was observed in 82% of patients in the first group. Morphometric changes in X-ray demonstrated inhibition of the destruction in articular cartilage and surfaces of bones after transplantation of fetal chondrocytes. The dynamics of morphological changes in synovium showed 2.5 times reduction of the inflammatory reaction. Transplantation of fetal chondrocytes led to a significant reduction in ESR, CRP, fibrinogen, γ-globulin after a period of 12 months (*p* < 0.03). Furthermore, patients in the second group had 2.7 times higher risk of ankylosis compared to the first group. We did not observe any complications of fetal chondrocytes transplantation.

**Conclusions:**

Application of fetal chondrocytes therapy had the desired clinical effect, which was confirmed by reduction of the RA activity and decrease of cartilage and bone destruction.

